# Exploring dimensions of blended learning readiness: Validation of scale and assessing blended learning readiness in the context of TVET Bangladesh

**DOI:** 10.1016/j.heliyon.2022.e12766

**Published:** 2023-01-02

**Authors:** Shariful Islam Shakeel, Md Faruque A. Haolader, Mst Sharifa Sultana

**Affiliations:** a Department of Technical and Vocational Education, Islamic University of Technology, Gazipur 1704, Bangladesh; b Bangladesh Institute of Marine Technology, Narayanganj 1410, Bangladesh

**Keywords:** Blended learning, Assessing readiness, TVET, Blended learning readiness scale, Online learning, Polytechnic students, TVET Librarians, Bangladesh

## Abstract

In collaboration with Technical and Vocational Education and Training educators and academic librarians, this study attempts to develop and validate a blended learning readiness scale for Bangladeshi TVET students. This study also attempts to investigate the reliability of this validated scale by measuring six blended learning readiness dimensions. In this research, the Content Validity Index, Exploratory Factor Analysis, and Confirmatory Factor Analysis were used to establish the construct validity of the blended learning readiness scale. Questionnaires were circulated to the students of six Institutes of Marine Technology (N = 235) who went for a blended learning session for an entire semester. This study's result suggests a strong positive association between Bangladeshi polytechnic students' preparedness for blended learning and attitudes toward online learning, openness to new technology, and attitudes toward the face-to-face classroom. In contrast, significant negative correlations were found between blended learning readiness and basic skills in using technology, learning flexibility, and study management. This study also implied that gender and previous academic achievement is not strong predictor of measuring blended learning readiness in Bangladeshi Technical and Vocational Education and Training context. This blended learning readiness scale would help course designers, educators, librarians, and policymakers of Bangladesh to improve the quality of the blended learning environment by addressing the students' concerns about various blended learning components.

## 1 Introduction

With the onset of the Industrial Revolution 4.0, the usage of technology has been gradually transforming the educational landscape. TVET institutions are working to improve the pedagogical approach that uses educational technology to empower students to create and manage their own learning experiences. Academic librarians are also looking for creative methods to better serve teachers and students in light of global technological progress [1]. The upsurge of COVID-19 has further fastened the process of embracing technology and starting online education. With the increased adoption of online learning concepts and methodologies by higher education institutions, the domains of learning and teaching are undergoing major changes [2]. In response to students' different requirements and aspirations and the need for additional time to meet expanding curricular demands, several higher education institutions have introduced online-only, hybrid, or blended learning courses [3].

In particular, the Department of Polytechnic Education and Community Colleges specifies that post-Covid'19 learning should be conducted in both online and face-to-face settings [4]. A study found that Bangladeshi youth mostly use smartphones and widespread 4G mobile access in joining blended learning sessions [5]. However, students who move to blended classrooms during the pandemic may have faced new challenges regarding online class preparation, participation, and activities [6]. Thus, opinions from organizations, instructors, course designers, librarians, and students are critical. Particularly, the student's perspective is crucial for successfully implementing blended learning in technical and vocational institutions. Few researchers call for more research on student attitudes toward blended learning, and few advocates measure learner readiness prior to fully implementing this unique teaching and learning approach [7,8]. Researchers have been working on the online and blended learning readiness scale for some years. Due to the growing popularity of online learning in educational institutions, course designers and librarians had to re-evaluate students blended learning preparation and construct a scale for evaluating student readiness that is more comprehensive. Thus, teachers can better design online courses and help students to ensure good online learning experiences by doing this.

It is vital to identify the dimensions of online learning preparation that college students should possess and those that may have been overlooked in previous studies to gain a better understanding of how to implement efficient online learning. Several pieces of research examined whether online learning preparedness was a significant predictor of student satisfaction in online courses [9]. The study suggests that online learning readiness is critical for motivating learners to participate in blended learning activities [10]. There is still much to learn about how learners perform in current blended learning settings that are supported by online/blended platforms. Furthermore, this study intended to fill a gap in the literature by assessing the readiness of Bangladeshi TVET students who took a blended course recently. This study focuses on students' perceived competency and comfort levels when working in a blended learning context. As per the eighth five-year plan (July 2020–June 2025) of Bangladesh, strategic guidelines are prepared to create a knowledge and skill-based society. The TVET graduates will play a pivotal role here in Bangladesh to shoot up the employment rate by 55% by 2030. Thus, it is important to know whether the TVET students of Bangladesh are comfortable using the new generation technology in their education in the midst of the pandemic. But due to the unavailability of a valid blended learning readiness scale (BLRS) in the context of TVET Bangladesh, this study endeavored to make one. In this study, both TVET educators and librarians collaborated to prepare a BLRS. Moreover, the students attended an entire semester of blended learning sessions before responding to the questionnaire to make this scale one of a kind. In this study, the researcher tried to explore dimensions of the blended readiness scale as well as evaluate and validate the scale specifically for upper secondary non-tertiary level TVET blended learning programs. This study attempted to answer the below two research questions:

RQ1 Could the validated scale be used to assess Bangladeshi TVET students' readiness toward blended learning?
RQ2 Does a student's preparation for blended learning depend in any way on their gender and previous academic performance?

## 2 Blended learning and TVET sector of Bangladesh

Blended learning is an educational approach that employs a variety of delivery methods to get the best possible learning outcomes while also keeping program costs as low as possible. However, It's crucial to concentrate on the outcomes of teaching and learning, not just to mix and match different learning delivery methodologies [11]. Students can benefit from blended learning if one blends the best elements of online material delivery with classroom interaction and live instruction to create an engaging learning environment that encourages thoughtful thinking and tailors training to meet the needs of a varied set of students [12]. Educational psychologists frequently view self-regulation as a prerequisite for successful learning in school and beyond [13]. Self-directed learning is contingent upon learners' capacity to activate and sustain their own ideas, activities, and emotions during the learning process in order to accomplish their objectives [14]. In higher education, blended learning is widely employed to provide more flexible learning paths. Students who use blended learning have more control over the timing, location, and pace of their studies. Students may also utilize online learning systems from any location at any moment that has an internet connection, not only in the classroom [15].

The development of a nation's intellectual human capital is greatly aided by technical vocational education and training (TVET). Several changes have taken place in TVET's mission to generate high-quality, highly competitive, and skilled human capital [16]. The goal of TVET is to enhance a person's ability to do a task through the provision of suitable training. Rural areas are home to the majority of TVET institutions in Bangladesh. In terms of education and learning, Bangladesh's rural and urban areas have large knowledge disparities [17]. These issues can be alleviated by using blended learning environments to narrow the learning gap between developed and underdeveloped areas. The COVID-19 epidemic has left Bangladesh in a grave state across the country. While the government enacted a lockdown strategy to maintain social distance and prevent virus outbreaks, the majority of Bangladeshi educational institutions failed to encourage student participation in online learning [18] due to the fact that the idea is relatively fresh to Bangladeshi learners [19]. Besides, lack of necessary devices, access to the internet, traditional mindset to engage in a new form of learning, lack of motivation for self-regulated learning, the interaction between student-student and teacher-student, lack of teacher training program, attitude towards new technology, all are the reasons for unsuccessful blended learning sessions [20]. A recent study also concurs that teachers in Bangladesh have low technology dependency as well as low technical skills to effectively adopt modern technology in teaching activities [21,22]. Therefore, Bangladeshi TVET experts could play a major role in this area of promoting blended learning by making the overall learning process student-friendly. Apart from TVET course designers and instructors, TVET librarians in Bangladesh should also make deliberate efforts to acquire abilities so that they can compete with their counterparts in other developed nations for a significant and major role in the ever-changing, sophisticated learning environment. There is no denying the practical significance of blended learning (BL) in Bangladeshi institutions, but the evidence implies that policy should be developed to address infrastructure needs, program development, and strategic planning [20].

## 3 Dimensions of blended learning readiness scale

People who want to accomplish a task must have the mental, physical, and cognitive preparedness required for their responsibilities. Students and teachers who serve as the focus of instruction in the learning-teaching activities should always be well-prepared for the events they will engage in during the course [23]. Readiness is a mindset of students who are prepared to engage in activities with full awareness to achieve outcomes in the form of changes in their comprehension of the subject matter, as well as in their abilities to apply that information, understanding, skills, habits, values, and attitudes. To be ready to learn, pupils must possess certain capacities relevant to a certain educational goal. Preparation can help students cope better in stressful situations. It's easier for them to comprehend issues and devise remedies. Studying how students react to online learning after it has been implemented in the classroom is an important part of the growing body of research on blended learning innovation. Additionally, this type of study also involves research that evaluates the perceived effects of adopting an invention before the innovation is implemented [24]. The ability to engage in self-directed learning, student preferences for online learning over the face-to-face classroom, proficiency, comfort in using the internet and computer-mediated communication, and other criteria can all be used to assess a student's preparation for online learning [2].

To concretely represent the notions of preparedness, McVay [25] designed a 13-item questionnaire to assess online learning readiness. The instrument's predictors include student behaviour and attitudes. Hung et al. [2] proposed that the online learning readiness measure contains online self-efficacy, learner control, desire for learning, self-directed learning, and self-efficacy in online communication. Dray [26] developed a survey design that focuses on two subscales, e.g., (learner characteristics and ICT engagement) to assess the readiness for online learning. According to Tang [10], there are six characteristics of learning that may be studied to determine a student's preparation for blended learning. These six facets of learning are attitude toward technology, online interaction, flexibility, attitude towards online learning, study management, and Face to Face classroom learning. In most of the previous studies, the authors attempted to develop a blended learning readiness scale (BLRS) without indulging respondents in any real blended learning sessions [10]. In order to overcome the drawback of the previous scale, the authors prepared an entire blended learning session for its respondents. A blended learning readiness framework of six components was also developed by the authors in this study. After the successful completion of an entire semester, the authors investigated those readiness dimensions to measure the reliability of this BLRS and assess the blended learning readiness of Bangladeshi TVET students.

### 3.1 Learning flexibility

Learning to be adaptable is the first step in assessing learning readiness. Students increasingly have to juggle school, employment, and family commitments; therefore, learning flexibility is becoming increasingly important [27]. The advantages of blended learning include saving students valuable time and allowing them to study whenever and wherever they choose [28]. Students view blended learning as a way that enables them to study at their own speed and in their own time and encourages them to develop greater self-reliance in their learning [29].

### 3.2 Openness to new technology

Technology delivers knowledge interactively to learners using text, video, simulation, and animation as examples of new alternatives for providing information [30]. It is now possible to use communication technology in educational settings. These technologies allow for subtle changes in synchrony. Blended learning is much more than just combining classroom instruction and online learning. This allows for a greater variety of learning scenario designs. IT supports blended learning, and digital tools assist in creating online communities that span borders and time zones. For blended learning to be successful, learners must have openness and easy access to digital tools [31].

### 3.3 Attitudes towards online learning

The third factor is the attitude toward online learning. An advantage of online learning is that students have more time to think about their answers before submitting them. Blended learning has the effect of bringing timid students out of their shells, as evidenced by the fact that most students converse more online than they do in a face-to-face setting [15]. Students who favour online education indicate that they have more time to consider and respond to asynchronous dialogues, according to previous research [10].

### 3.4 Attitude towards face-to-face classroom

The fourth part of assessing is the attitude toward Face-to-Face classroom instruction. There may be differences in the theoretical foundations and educational goals of online instructors, just as there are with traditional onsite classrooms. Many students believe that face-to-face interactions are better suited to activities that require students to investigate and engage in problem-solving. A study by Stodel et al. [32] suggests online forums often fail to foster dynamic discussion and match the sensation of a real conversation. When it comes to students withdrawing from online courses, it is found that those who crave face-to-face engagement with their peers and lecturers are more inclined to do so [33].

### 3.5 Basic skills in using technology

The fifth part of assessing readiness is the basic skills of using technology. A study by Mijatovic [34] found that different forms of interactions with online technology might lead to varying degrees of learning results. According to Harris [31], discussion and interaction are crucial components of learning and should be integrated into a blended learning setting. Asynchronous Web-based discussion forums can be used for open communication or critical debate [3]. However, if the students lack basic skills in using these technologies, it will hamper the overall learning environment. Successfully blended learning sessions need participants to have basic skills and knowledge about computers and current ICT tools and platforms. Some individuals experience fears when utilizing computers and other modern ICT [35].

### 3.6 Study management

The sixth element of assessing learning preparedness is called “study management.” An independent learning process in which students organize, manage, and coordinate their own learning activities and collaborate with their teachers [36]. As a result, students who are taking classes online will have an easier time keeping track of their time and staying motivated. Blended learning empowers students to take charge of their own education, which necessitates self-discipline and self-inspiration. With the help of online and face-to-face classes, students in blended learning programs may learn at the pace that best suits their schedules [37].

## 4 Methodology

The ethical approval for this study was obtained from the Committee for Advanced Studies and Research (CASR) of the Islamic University of Technology (IUT) in its 50th meeting (Ref. No. CASR/50/2022/01/Proc/001) on March 28, 2022. The authors confirm that all the people who participated in the study from the six Institutes of Marine Technology (IMT) gave informed consent.

### 4.1 Survey instrument

A survey was utilized to gather data for this study, which is a quantitative one. Using a survey was deemed acceptable since it enabled researchers to analyze each of the readiness factors of blended learning components independently and their links to each other [38]. Researchers utilized a survey to find out how students felt about various aspects of blended learning. A blended learning course named “IC Engine Principle” was developed by the authors to indulge students in a real blended learning environment. The course was delivered by the authors in collaboration with the instructors of six Institutes of Marine Technology. A total of 240 1st semester marine technology students from all six polytechnic institutes under the Bureau of Manpower Employment and Training (BMET) were engaged in the Blended Learning course. After the completion of the semester, data was collected from 235 students out of 240 students. Six TVET experts were engaged in the item development and validation process to ensure no critical items of blended learning remained untouched. A survey questionnaire using a four-point Likert scale was developed to record the response of the TVET students. Where 4 indicated “strongly agree” and 1 indicated “strongly disagree."

### 4.2 Content validity instrument development

In this study, authors aimed to construct and validate the blended learning readiness scale that accurately represents items' content domain. The authors selected items for integration in this tool based on Content Validity Index (CVI) and Scale Level Content Validity Index (S-CVI). S-CVI is a vital early step in improving the construct validity of a survey instrument or questionnaire. In order to demonstrate that the scale and the items on it are valid in terms of content, developers of new scales are increasingly expected to present evidence. The researcher conducted a review of existing literature as part of their investigation [39] [40] [41] to develop the Content Validity Index (CVI) scale. CVI is a metric that assesses the degree to which experts agree on a subject's content. In this study, TVET experts were given the objectives and items and asked to rate the relevance of each item in connection to the objective(s) on a scale of 1–4: (1) not relevant, (2) somewhat relevant, (3) quite relevant, and (4) very relevant.

### 4.3 Model validation and hypothesis development

**Fig. 1:**
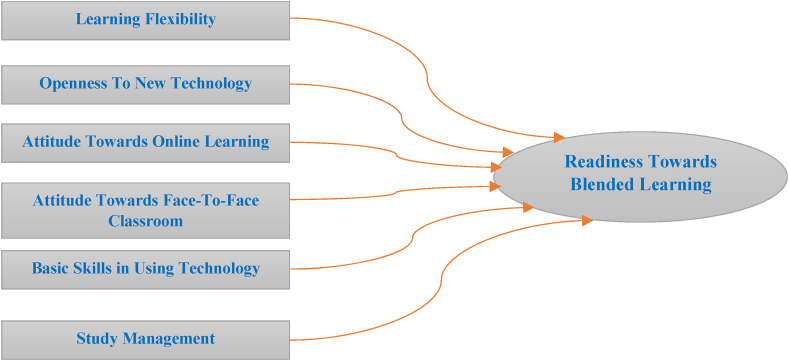
Theoretical framework of exploring dimensions of blended learning readiness scale.

After reviewing the cognate literature and having a discussion on the readiness aspect towards a Blended Learning environment, this study has come up with the following blended learning readiness constructs to determine the blended learning readiness of Bangladeshi TVET students. Appendix A shows the underpinning items under each construct that were revised after conducting the content validation using the Content Validity Index (CVI). EFA was used to explore the underpinning items of each construct to determine the readiness scale for a Blended Learning environment. Due to the unobservability of latent constructs, they are frequently represented by items. According to the principle of unidimensionality, each item must represent a single latent construct. This study used structural equation modelling (SEM) based on partial least squares (PLS) [42]. PLS is an excellent technique for predictive applications. Alternatively, the PLS method is ideal for generating predictions since it examines variances and the importance of associations. PLS analysis utilizes two types of models: measurement and structural. PLS offers a CFA by analyzing both models. The structural model, also known as the inner model, describes the links between the exogenous and endogenous latent constructs, whereas the measurement model, also known as the outer model, depicts the latent constructs and their items [42]. Hair et al. [43] presented numerous suggestions to put the measurement and structural model to the test. It is suggested to establish the convergent validity of the measurement model, also known as an outer model, using the outer loadings of the survey questions and the average variance extracted (AVE). The Fornell-Larcker criterion and cross-loading were employed to assess discriminant validity. Additionally, Henseler et al. [44] recommended analyzing the Heterotrait-Monotrait relationship in order to determine tighter discriminant validity. It is also recommended to use the bootstrapping approach to determine the importance of route coefficients based on t-values [43]. The theoretical underpinnings of measuring the reliability of this blended learning readiness scale are depicted in Fig. 1.

Six hypotheses were developed for this study after studying the extant literature, and the responses indicated how reliable the validated model is for determining if Bangladeshi TVET students are prepared for blended learning.

H1 *Students' attitude towards learning flexibility has a positive influence on being engaged in blended learning.*
H2 *Students' openness to (new) technology positively influences engagement in blended learning.*
H3 *Students' attitude toward online learning has a positive influence on being engaged in blended learning.*
H4 *Students' attitude toward face-to-face classrooms has a negative influence on being engaged in blended learning.*
H5 *Students' basic skill in using technology has a positive influence on being engaged in blended learning.*
H6 *Students' attitude* toward *study management has a positive influence on being engaged in blended learning.*

### 4.4 Participants

This study comprises two groups (Table 1). Six expert reviews were sought to validate the instrument. One of them was a professor with a specialization in Educational Technology, three were senior instructors/instructors, and two were librarians from different TVET institutes in Bangladesh. All the reviewers had a handful of experience in designing and conducting online courses. Six TVET institutes' 1st-semester marine technology students (N = 240) had undergone a blended learning course specially designed for them and eventually responded to the questionnaires validated by reviewers.

**Table 1:** Demographic information of the respondents.

Demographic Information	Respondents (N = 235)	Number	Percentage
Gender	Male	209	89%
	Female	26	11%
Device	Smartphone	212	90.2%
	Computer	10	4.26%
	Laptop	10	4.26%
	Tablet	2	0.85%
	No Device	1	0.43%
Internet Access	Yes	180	77%
	No	55	23%
Capable to Navigate through webpages	Yes	220	93.6%
	No	15	6.4%
Capable of downloading and uploading files	Yes	223	94.9%
	No	12	5.1%
Participation in Quiz/Assignment (at least once)	Yes	151	64.26%
	No	84	35.74%

Table 1 shows that 235 respondents were used to evaluate the reliability of the proposed BLRS and measure the readiness of Bangladeshi TVET students. Female respondents accounted for just 11% of all responses, indicating that the proportion of women admitted to polytechnic institutes is still below the national average. 90% of the 235 respondents had smartphones, which is a significant number. Twenty-three percent of the respondents did not have access to the internet, and 6.4% had difficulty navigating through web pages. There were 5.1% of those who were unable to download or upload files. There was around 36% of students did not attempt any quiz/assignment in that whole semester. In this study, it turned out that there were three students who didn't have any kind of email address at all.

## 5 Results

### 5.1 Validation of the items of blended learning readiness scale

In this study, evidence and best practices were used to develop the readiness scale as well as quantify the content validity index. To assess the readiness of Bangladeshi TVET students, the authors articulated 42 items for this survey instrument of respondents. In the end, the BLRS consisted of 29 items (**Appendix A**), each of which is inferred from I-CVI values. The item is deemed acceptable if its score exceeds 79%, requires adjustment if its score falls between 70% and 79%, and must be discarded if its score falls below 70% [45]. A CVI of 0.80 or greater is considered excellent content validity [39]. On this scale, all the items I-CVI were greater than 0.800. The items which fall below the desired value were ultimately discarded. The S-CVI/UA and S-CVI/AVE of the items under each construct were greater than 0.80, failing which were subjected to elimination. As shown in Table 2, the mean S-CVI/UA and S-CVI/AVE were greater than 0.800, which confirms the instrument's validity. SPSS was used to determine the Cronbach alpha coefficient, a measure of an item's reliability. Cronbach's Alpha is a measure of reliability that relates to the variation assigned to the construct's final scores. Inter-item correlation is used to determine if the domain was accurately assessed by the produced items and to demonstrate homogeneity. This rating must be as high as feasible, as low levels indicate a lack of reliability and will be discarded [46]. Items' reliability was greater than 0.700 and deemed acceptable [47,48].

**Table 2:** Content Validity Index (CVI) of the items.

Constructs	Items Number	Mean S-CVI/AVE	Mean S-CVI/UA	Cronbachs' Alpha
Attitude toward Learning Flexibility (LF)	3	0.889	0.833	.750
Openness To (New) Technology (NT)	5	0.867	0.833	.733
Attitude Toward Online Learning (OL)	5	0.889	0.825	.804
Attitude Toward Face-To-Face Classroom (CL)	5	0.958	0.833	.714
Basic skills in Using Technology (BST)	4	0.917	0.833	.725
Attitude Toward Study Management (SM)	3	0.944	0.825	.834
Readiness Toward Blended Learning (BL)	4	0.958	0.833	.897
**Total Mean S-CVI/AVE**		0.917		
**Total Mean S-CVI/UA**			0.831	
**Mean Cronbach's Alpha**				0.779

### 5.2 Exploratory Factor Analysis (EFA)

EFA is a tool to recognize the number of items in each construct. The study aims to investigate individual items across variables and categorize each component based on high inter-item correlations [49]. Items that have a high correlation with one component but not with others are put together to create a scale, and any item that has a cross-load on many factors or a weak load on any factor is subject to elimination [50]. In EFA, principal axis factoring is one of the most often used estimate approaches. It is commonly recognized that Principal Axis Factoring improves the recovery of weak factors [51]. The KMO sample adequacy score was 0.728, above the recommended threshold of 0.60 [52], and Bartlett's test was significant (p = 0.000), indicating that the correlations between questions were statistically significant for the question analysis [53]. In this study, analyses were initially done to exclude any items with an inter-item correlation of less than 0.4 [54]. In this analysis phase, we had to eliminate five items out of the twenty-nine, as they were either loaded with other constructs or had a load factor of less than 0.4. Following item analysis using Principal Axis Factoring, this study left with a total of 24 items (Table 3). Six constructs accounted for 60.65% of the total variance. Table 3 summarizes the items loaded on the various criteria. Cronbach's alpha values for all factors were greater than the cutoff of 0.7 and were deemed satisfactory [52]. The construct attitude towards study management was abolished when its components were eliminated or shifted to other variables. As an outcome, hypothesis **H6** was not supported.

**Table 3:** Summary of factors using principal axis factoring.

Construct	Items	Mean	Std. Dev.	LF	NT	OL	CL	BST	BL
Learning Flexibility (LF)	I would like to access the class materials on my own time	3.281	0.618	.498					
	I would like to access the class materials at my own pace	3.285	0.647	.825					
	I would like to access the class materials in my own space	3.374	0.644	.795					
Openness to new technology (NT)	I prefer technology while learning	3.557	0.531		.522				
	Access to all digital learning materials helps me understand my lesson Precisely	3.349	0.815		.767				
	I would like to be in charge of my learning	3.498	0.682		.518				
	I would like to keep myself updated with new educational technology	3.553	0.507		.560				
	I believe technology improves my quality of learning	3.557	0.515		.521				
Attitude toward online learning (OL)	I believe online tasks help me build my learning capacities	3.166	0.807			.475			
	I am comfortable with self-directed online learning	2.855	0.899			.785			
	I do not feel isolated in an online classroom	2.609	0.867			.707			
	I believe online learning helps me to prepare well for my future endeavours	2.885	0.867			.634			
	I prefer an online platform to communicate with other teachers and students	2.991	0.847			.643			
Attitude toward Face-To-Face Classroom (CL)	I prefer to learn in the face-to-face classroom environment	3.762	0.456				.710		
	I learn better in a teacher-directed face to face classroom	3.745	0.447				.727		
	I believe face-to-face learning develops my interpersonal and team-building skills	3.757	0.439				.624		
	I prefer immediate feedback from the teacher	3.630	0.542				.624		
	I prefer learning through collaboration with other people in Face-To-Face Classroom	3.617	0.513				.535		
Basic skills in using technology (BST)	I have basic skills in using technology	3.379	0.625					.526	
	I can understand the online instruction for assignments/quizzes/tutorials	3.170	0.617					.606	
	I can manage unwanted situations and download the learning materials	3.243	0.582					.569	
Readiness towards Blended Learning (BL)	I am more comfortable with blended learning than face-to-face learning	2.740	0.890						.526
	I want to attend courses that offer blended learning	3.149	0.640						.772
	I believe a blended learning environment improved my learning capacity	3.098	0.706						.605
	**% of variance explained**			17.713	13.657	8.271	7.693	7.106	6.211
	**Eigenvalue**			4.251	3.278	1.985	1.846	1.705	1.491
	**Cronbach's alpha**			.746	.724	.800	.710	.704	.810

### 5.3 Confirmatory factor analysis (CFA) and assessing blended learning readiness of Bangladeshi TVET students

The reliability and convergent validity of the readiness model were determined using item-level PLS factor loadings, average variance extracted (AVE), and composite reliability (CR). Only those items that exceeded the recommended composite reliability (0.70) values for confirming the measurement model's reliability were preserved during this phase. As seen in Table 4, twenty-two items value satisfied the required average variance extract cutoff value of 0.50. Thus, the model's constructions were all sufficiently reliable and valid [43]. One item from learning flexibility and one item from the readiness towards blended learning were dropped as the factor loading was below 0.40 [55]. When the AVE is less than 0.50, and the composite reliability is greater than 0.60, the construct's convergent validity is still acceptable [56]. Also, in this study, the values of Cronbach's alpha were found to be greater than 0.7 and deemed satisfactory [52].

**Table 4:** Reliability, AVE, CR of confirmatory factor analysis.

Measures	Items	Cronbach's alpha	Composite Reliability	Average Variance Extracted
Learning Flexibility	2	0.835	0.904	0.826
Openness to new technology	5	0.742	0.824	0.486
Attitude toward online learning	5	0.783	0.850	0.535
Attitude toward face-to-face classroom	5	0.786	0.832	0.505
Basic skills in using technology	3	0.714	0.765	0.535
Readiness toward blended learning	2	0.705	0.852	0.745

Individual Item Reliability measures how well multiple-item scale measurements represent the real score of the latent variables compared to the error. Table 5 shows that all variable loadings are larger than 0.40 and deemed satisfactory. Moreover, collinearity influences weight estimations and statistical significance. As a result, it is tougher to establish that the projected weights are meaningfully different from zero. Unreliable weight estimates and sign reversal are also possible. Collinearity is analyzed in PLS-SEM using the Variance Inflation Factor (VIF). The variance inflation factor (VIF) values in Table 5 are all less than 5.0, suggesting that the model is not multicollinear [43].

**Table 5:** Factor loading matrix.

Constructs	BL	BST	CL	LF	NT	OL	VIF
Readiness towards Blended Learning (BL)							
BL2	0.750						1.421
BL3	0.963						1.421
Basic skills in using technology (BST)							
BST1		0.478					1.163
BST2		0.905					1.243
BST3		0.747					1.305
Attitude toward face-to-face classroom							
CL1			0.696				1.639
CL2			0.933				1.831
CL3			0.601				1.449
CL4			0.662				1.323
CL5			0.607				1.384
Learning flexibility (LF)							
LF2				0.986			2.059
LF3				0.825			2.059
Openness toward new technology (NT)							
NT1					0.715		1.318
NT2					0.825		1.515
NT3					0.611		1.250
NT4					0.649		1.426
NT5					0.665		1.404
Attitude toward online learning (OL)							
OL1						0.589	1.245
OL2						0.813	1.867
OL3						0.778	1.678
OL4						0.769	1.525
OL5						0.684	1.507

Fornell-Larcker suggested that to show discriminant validity, the square root of AVE must be higher than the constructs' correlations with all other structural model constructs [57]. It is clear from Table 6 that each indicator's cross-loading exceeds the underlying constructs own cross-loading, which demonstrates the validity of the measurement model used in this work.

**Table 6:** Latent variable correlations among constructs using the Fornell-Larcker criterion.

Construct	CL	OL	BST	LF	NT	BL
Attitude toward the face-to-face classroom (CL)	0.710					
Attitude toward online learning (OL)	−0.088	0.731				
Basic skills in using technology (BST)	0.184	0.002	0.731			
Learning Flexibility (LF)	0.005	0.123	0.062	0.909		
Openness to new technology (NT)	0.209	0.202	0.292	0.297	0.697	
Readiness toward blended learning (BL)	0.105	0.264	0.176	0.076	0.331	0.863

The HTMT is a metric used to compare latent variables. Discriminant validity can be regarded as established if the HTMT is evidently less than 1. A cutoff of 0.85 consistently distinguishes latent variable pairs that are discriminant valid from those that are not in various real-world settings [44]. Table 7 depicts that all construct values in the model are less than the threshold value of 0.85, indicating that the model has appropriate discriminant validity.

**Table 7:** Results of Heterotrait-Monotrait (HTMT) Ratio for discriminant validity.

Construct	CL	OL	BST	LF	NT	BL
Attitude toward the face-to-face classroom (CL)						
Attitude toward online learning (OL)	0.226					
Basic skills in using technology (BST)	0.348	0.209				
Learning Flexibility (LF)	0.124	0.201	0.150			
Openness to new technology (NT)	0.371	0.293	0.468	0.373		
Readiness toward blended learning (BL)	0.107	0.302	0.193	0.086	0.374	

**Fig. 2:**
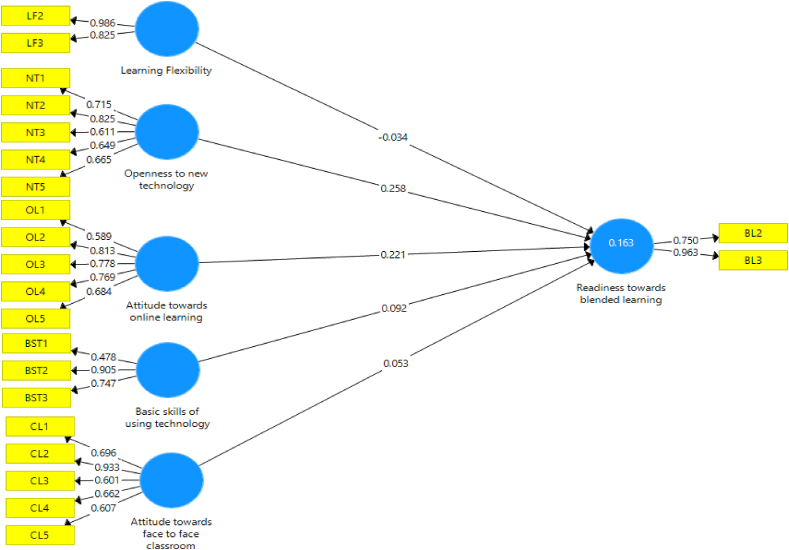
Results of the structural mode.

The predictive explanatory power (R^2^ value) and cross-validated redundancy (Q^2^ value) of the structural model have been checked using the PLS-SEM (Fig. 2). R^2^ of variables reflects the extent to which they effectively explain the dependent variables (see Table 8). Cohen [58] advised that when R^2^ values are respectively 0.26, 0.13, or 0.02, predictive explanatory power could be categorized as significant, moderate, or weak. This model demonstrates a weak explanatory power (R^2^ = 0.049). Additionally, the authors assessed the model's predictive relevance for the latent dependent variables using Q^2^. It is widely used to assess a model's predictive relevance and may be accomplished using the blindfolding method provided in the majority of PLS software solutions. If Q^2^ is greater than 0, the model is regarded as predictively significant [59,60]. The structural model is acceptable based on the data in Table 8 since the exogenous constructs have predictive relevance for the model's endogenous components. PLS-SEM model fit can be measured with Standardized Root Mean Square Residual (SRMR). In this study, Smart PLS yields an SRMR value of 0.068 (Table 8), which is less than the threshold value of 0.08 [61], indicating that the overall model of this study exhibits a reasonable degree of adaptability [62].

**Table 8:** Results of structural model.

Constructs	Predictive explanatory power (R^2^)	Cross-validated redundancy (Q^2^)	SRMR
Readiness toward blended learning	0.049	0.064	0.068

The hypotheses test result (Table 9) reveals that hypothesis ***H2*** attitude toward online learning (β = 0.203, t = 2.355, p < 0.05) and **H5** openness to new technology (β = 0.221, t = 2.653, p < 0.05) have a statistically significant influence on readiness towards blended learning. Thus hypotheses ***H2*** and ***H5*** are supported. But hypothesis ***H1*** – attitude towards face-to-face classroom learning (β = 0.071, t = 0.943, p > 0.05), Hypothesis ***H3*** – basic skills in using technology (β = 0.1, t = 1.307, p > 0.05) and, ***H4*** – learning flexibility (β = 0.035, t = 0.287, p > 0.05) do not have a statistically significant impact on readiness towards blended learning. Thus, ***H1***, ***H3***, and ***H4*** do not support the hypothesis. Cohen [58] defined f^2^ values of 0.02, 0.1, and 0.35 as indicating small, medium, and large impact sizes, respectively. All the hypothesis in this study has a small effect size. But Cohen's benchmarks shouldn't be used as sole generic impact size descriptors. Cohen's labels may be misleading because certain domains, like education, have lesser effect sizes [63]. Bakker et al. [64] also state that, When interpreting effect sizes, sample size sometimes plays an important role.

**Table 9:** Results of Hypotheses testing using path analysis.

Hypothesis	Relationship	Std. beta (β)	Std. dev.	t-value	P-value	decision	f^2^
***H1***	CL **→** BL	0.053	0.086	0.617	0.538	Not supported	0.003
***H2***	OL **→** BL	0.221	0.064	3.463	0.001	Supported	0.055
***H3***	BST **→** BL	0.092	0.081	1.142	0.254	Not supported	0.009
***H4***	LF **→** BL	−0.034	0.079	0.427	0.670	Not supported	0.001
***H5***	NT **→** BL	0.258	0.062	4.167	0.000	Supported	0.062

### 5.4 Gender difference in blended learning readiness

To ascertain whether there is a difference in Blended Learning Readiness between males and females, a one-way MANOVA was performed. This test took into account blended learning readiness scale dimensions. Table 10 depicts that there was no significant difference between males and females on the blended learning readiness measures.

**Table 10:** Descriptive statistics and F test of gender on Blended Learning Readiness dimensions.

Dimension	Gender				F	P	Partial eta squared
	*Male*		*Female*				
	*M*	*SD*	*M*	*SD*			
Attitude Toward Learning Flexibility (LF)	3.311	0.543	3.333	0.365	0.042	0.839	0.000
Openness To (New) Technology (NT)	3.501	0.423	3.523	0.503	0.063	0.802	0.000
Attitude Toward Online Learning (OL)	2.892	0.642	2.977	0.519	0.422	0.517	0.002
Attitude Toward Face-To-Face Classroom (CL)	3.723	0.336	3.639	0.426	0.506	0.411	0.004
Basic skills in Using Technology (BST)	3.276	0.469	3.167	0.330	1.327	0.251	0.006
Attitude Toward Study Management (SM)	3.234	0.420	3.218	0.461	0.035	0.852	0.000
Readiness Toward Blended Learning (BL)	3.002	0.575	2.949	0.522	0.199	0.656	0.001

### 5.5 Grade difference in blended learning readiness

A one-way MANOVA was carried out to see whether there was a difference in Blended Learning Readiness among students who had exceptional academic grades, good academic grades, and average academic grades in the previous examination. A Grade Point Average (CGPA) greater than 4.5 out of 5.00 was considered excellent, a CGPA of 4.00–4.49 was deemed a good academic grade, and a CGPA lower than 4.00 was considered an average grade. No students had a CGPA of less than 3.5 in these six institutes of marine technology. Blended learning preparedness based on prior academic grades was not statistically different, as shown in Table 11. Thus, this study concluded that there were no statistical differences among students with outstanding, good, and average grades on integrated blended learning readiness.

**Table 11:** Descriptive statistics and F test of academic grades on Blended Learning Readiness dimensions.

Dimension	Previous Semester Academic Grades						F	P	Partial eta squared
	*Excellent*		*Good*		*Average*				
	*M*	*SD*	*M*	*SD*	*M*	*SD*			
Attitude Toward Learning Flexibility (LF)	3.474	0.374	3.417	0.477	3.383	0.292	.254	.777	.008
Openness To (New) Technology (NT)	3.514	0.526	3.463	0.550	3.420	0.420	.208	.813	.006
Attitude Toward Online Learning (OL)	2.890	0.750	2.779	0.689	2.632	0.657	.669	.516	.020
Attitude Toward Face-To-Face Classroom (CL)	3.750	0.316	3.726	0.396	3.710	0.386	.074	.929	.002
Basic skills in Using Technology (BST)	3.456	0.524	3.393	0.454	3.217	0.450	1.376	.260	.041
Attitude Toward Study Management (SM)	3.246	0.398	3.200	0.396	3.095	0.647	.529	.592	.016
Readiness Toward Blended Learning (BL)	3.100	0.659	3.070	0.562	2.905	0.543	.790	.458	.024

## 6 Discussion

### 6.1 Blended learning readiness scale

This study examines the validity and reliability of a blended learning readiness scale, which can facilitate research in this area and provides a conceptual framework for comprehending learner readiness in a blended learning environment. This research verifies that all the items on the instrument are acceptable to assist students in evaluating their preparation for blended learning by comparing their responses to those on the instrument. The findings of this research pointed to the fact that the instrument possesses significant degrees of reliability as well as validity. When it comes to reliability, it has been demonstrated that the instrument has high values of internal consistency as measured by Cronbach's alpha. The findings also provide a lot of cause for optimism regarding the reliability of the instrument. CVI reveals that every item possessed a greater value for both the I-CVI and the S-CVI, which was greater than 0.80 and deemed satisfactory [39]. Later, EFA ensured that all items were heavily loaded in each construct. Finally, confirmatory factor analysis confirmed the connections between the exogenous and endogenous latent constructs. Every single construct demonstrates sufficient levels of both reliability and discriminant validity. All six subscales' contributions to the composite reliability were above the necessary minimum of 0.70 [65]. All the factor loadings were significant (p < 0.001), which indicates that each item was accurately represented by the factors. As a result, it was found that the blended learning readiness scale is a reliable tool for assessing the attitude and conduct of blended learners.

### 6.2 Blended learning readiness of Bangladeshi TVET students

This study attempts to measure the preparedness of Bangladeshi TVET students toward blended learning using this proposed BLRS. Six hypotheses were analyzed with CFA using PLS-SEM and came with a mixed result.

#### 6.2.1 Blended learning readiness vs. learning flexibility of Bangladeshi TVET students

The first hypothesis states, “Students' attitude towards learning flexibility has a positive influence on being engaged in blended learning.” This hypothesis statement is not supported. Few previous studies reported that learning flexibility is a strong predictor of measuring blended learning readiness and should have a strong positive correlation between them [10,66]. This proposed BLRS implied that the TVET students of Bangladesh lack the quality of learning flexibility to be engaged in blended learning. Flexibility in learning is enabled through the creation of a computer-generated learning environment, which eliminates the constraints of location and time [67]. E-learning gives institutions, as well as their students or learners, greater flexibility in terms of the time and location at which learning material is delivered or received, depending on the content being learned. But, the majority of TVET students in Bangladesh do not have a separate room, which is also a hindrance to paying attention in online classes during lecture time [6]. According to the findings of this study, TVET students are not yet flexible enough to begin a blended learning program. The lack of resources during the epidemic is another significant impediment to their ability to be flexible in their approach to blended learning. Only 20% of the students had access to a computer or a laptop computer. The rest were entirely dependent on telephones or smartphones. Thus, their involvement with technical resources is below par may be seen in this statement. According to a study, the problem of unreliable internet connection during class time, the absence of adequate equipment, apathy in online education, and power outages are some of the reasons that need to be taken care of in Bangladesh [68]. Few extant literatures [6,68] conform with this study's findings, which suggests Bangladeshi students lack the quality of being flexible. Thus, this BLRS could be considered a reliable instrument for measuring the blended learning readiness of Bangladeshi TVET students.

#### 6.2.2 Blended learning readiness vs. openness to a new technology of Bangladeshi TVET students

The second hypothesis states, “Students' openness to (new) technology has a positive influence on being engaged in blended learning.” This hypothesis is supported in this research, which indicates that TVET students of Bangladesh have a positive attitude toward starting blended learning. This result is congruent with a few other studies that affirm the positive relationship between blended learning readiness and openness to new technology [69,70]. This study reveals that students' attitude toward technology does not appear to be a major worry when deciding whether or not to begin a blended learning course. The adoption of blended learning may be hindered by technological issues such as broadband internet access and computer skills [71], yet today's technologically aware generation of students seems unconcerned about such issues. The findings revealed a significant favourable association between online contact and technology, as well as openness for blended learning. This study uncovered the fact that over 90% of students had smartphones, and 9% of pupils owned a PC or laptop. This is indicative that students are pretty familiar with social media (Facebook, Twitter), search engines, web video (e.g., YouTube), and text chat; however, they are less familiar with tools such as wiki, forums, video chat, MS Office applications, and blog according to the results of the study. Several studies have reported similar results earlier on students' positive attitudes toward technology and their achievement in a blended learning setting [72]. Thus, this BLRS was an accurate scale to measure Bangladeshi TVET students' readiness.

#### 6.2.3 Blended learning readiness vs. attitude towards online learning of Bangladeshi TVET students

The third hypothesis states, “Students' attitude towards online learning has a positive influence on being engaged in blended learning.” This hypothesis is supported in this research, which indicates that TVET students in Bangladesh are prepared to start blended learning. In contrast to feelings of isolation and boredom, an online environment defined by a sense of belonging can be advantageous in fostering positive attitudes toward online learning in a blended learning environment [73]. The results show that students' positive views toward online learning are positively correlated with their online interactions with classmates and teachers in terms of having access to instructional content and taking ownership of their own learning. Additionally, the findings demonstrated a positive association between the online environment and preparedness for mixed-learning environments. Several studies [3,33,36] reported similar results earlier and found a positive relationship between attitude towards online learning and blended learning readiness. Thus, this BLRS is suitable to use in the Bangladeshi TVET context.

#### 6.2.4 Blended learning readiness vs. attitude towards face-to-face classroom of Bangladeshi TVET students

The fourth hypothesis states, “Students' attitude towards face-to-face classrooms has a negative influence on being engaged in blended learning.” This hypothesis statement is not supported. That means the TVET students of Bangladesh are positive to be engaged in blended learning. There is a negative correlation between attitudes toward traditional classroom learning and readiness for blended learning. According to Stodel et al. [32], first-semester students' lack of conversational literacy and leadership skills may prevent online discussion threads from being as conducive to critical thinking, vibrant talks, and introspective discussions as face-to-face discussions. But our study suggests Bangladeshi first-semester TVET students have a very positive mindset towards blended learning, and they want to engage themselves in blended learning more for their academic achievement. In the real blended classroom, around 90% of the students joined the blended learning session, though they were not forced to join the classroom and were not provided with any technical support. The result of this study is congruent with the literature of Tang [10]. Therefore, this proposed BLRS scale has successfully measured that Bangladeshi TVET students are positive about attending online classrooms.

#### 6.2.5 Blended learning readiness vs. Bangladeshi TVET students' basic skill in using technology

The fifth hypothesis states, “Students' basic skill towards using technology has a positive influence to be engaged in blended learning.” This hypothesis statement is not supported. That means the TVET students of Bangladesh must lack basic skills in using technology to be engaged in blended learning. For students who lack basic technological literacy, this might have an especially negative impact on their achievement in an increasingly digital world [33]. Our study suggests only ten percent of the students were found to have access to a laptop or computer. Despite their openness to new technologies, they have few tools with which to hone their skills using the most up-to-date technologies. Because most students don't know how to type or upload files to Google Classroom, they weren't able to tackle the project in its entirety. Only 210 students out of 235 students attended both the online and face-to-face classes. Rest was either absent or only joined the face-to-face class. Only 64% of the students attempted the quiz/assignment because few of them were unable to navigate through the classroom alone. This study's data support that Bangladeshi TVET students lack basic technology skills, and this BLRS is a suitable instrument to measure blended learning readiness.

#### 6.2.6 Blended learning readiness vs. attitude towards study management of Bangladeshi TVET students

The sixth hypothesis states, “Students' attitude towards study management has a positive influence on being engaged in blended learning.” This hypothesis statement is not supported. This result is not congruent with some other studies [27,33,36,71], which suggested a positive relationship between study management and blended learning readiness. As effective study management ultimately influences individual behaviour, students must manage their own behaviour in order to participate in blended learning, which has a positive effect on their ability to pace and direct their own learning. The construct “study management” no longer existed as items loaded on other factors during EFA. It indicates that study management is not a valid construct to measure Bangladeshi TVET students' readiness for blended learning. That means the TVET students of Bangladesh lack the study management skills to be engaged in blended learning.

### 6.3 Bangladeshi TVET students' readiness for blended learning and gender differences

The second research question explores whether a student's readiness for blended learning is influenced by their gender in any manner. The study's findings indicate no gender disparities. This result is in line with the previous study, which also found that there is no correlation between gender and blended learning readiness, which indicates both male and female students demonstrated comparable levels of preparedness across all readiness characteristics. Similar results were found by Bunz et al. [74], Chung et al. [75], and Hung et al. [2], who found that there didn't seem to be a gender difference in computer proficiency or willingness to learn with digital tools. The results of the current study, however, differ from those of Rafique et al. [76], who depicted that, based on the gender of the students, relationship analyses revealed a substantial difference in opinion on aspects of online learning preparedness. Male students were perceived more favourably than female students on some criteria. A recent study concludes that there are no significant differences between males and females regarding online learning outcomes [77]. Thus, this BLRS can be considered a handy instrument to measure blended learning readiness irrespective of gender.

### 6.4 Bangladeshi TVET students' readiness for blended learning and grade differences

The second research question also investigates if academic performance in any way affects a student's preparation for blended learning. According to the findings of the study, students' grade levels had no effect on how well they were prepared for blended learning. It is found that there is no correlation between academic grades and blended learning readiness. Students with excellent, good, and average academic grades demonstrated comparable levels of preparedness across all blended learning readiness components. This result is not congruent with previous studies, which found a correlation between student readiness and results. Sriwichai [69] and Winarso [23] depicted in their studies that students who were more prepared for online learning had better results, whereas those who were not prepared struggled or felt discouraged while studying and had lower academic ratings. Hung et al. [2] also discovered that there appears to be a correlation between students' grade levels and their preparation for online classes. The possible reason behind the incongruency with the previous result could be the aspired x-factor for Bangladeshi TVET students and educators. The relatively new teaching methodology might be the first factor. As blended learning is a relatively new concept for Bangladesh TVET students, all the students, irrespective of prior academic achievement, might be the same excited or fearful about the new learning dimension. The second factor could be their approach toward digital learning tools and materials. This outcome could be supported by the fact that TVET students generally encounter the same technological challenges and limitations throughout their coursework, and this BLRS could be used for all TVET students irrespective of their previous academic performance. Nevertheless, this discovery may give academics new opportunities to investigate this phenomenon in more depth.

## 7 Conclusion

The increasing use of blended learning in educational institutions to improve teaching and learning has made it necessary to assess student's level of preparedness for participating in it. This study successfully validated an instrument that is reliable enough to measure blended learning readiness effectively in the Bangladeshi TVET context. The BLRS has a high Content Validity on individual items (I-CVI ≥0.80) and also has a high Scale Level Content Validity (S-CVI/UA = 0.831, S-CVI/AVE = 0.917). This BLRS also turned out to be a reliable scale to measure blended learning readiness. Thus, using the scale, researchers were able to address a number of concerns that need to be taken care of to improve the Bangladeshi TVET students' preparedness toward blended learning. These issues must be solved before students may successfully participate in blended learning. This study shows that students' knowledge and preparedness in many elements of blended learning must be improved and expanded upon to adapt to the blended learning environment. This BLRS indicates that student preparation to participate in blended learning varies from segment to segment, with lower readiness for some and higher for others. This scale discovered that students' preparedness for learning flexibility, study management, and basic technological abilities do not help them in being fully involved in blended learning to its full potential. This scale also found that Bangladeshi TVET students have a favourable attitude toward new technology, have demonstrated an excellent reaction to online learning, and their approach towards face-to-face classroom indicates a favourable mindset for engaging in the blended learning environment. This research might assist us in identifying the underlying components that are required to prepare Bangladeshi TVET students for a mixed learning environment in the future.

The authority must consider students' views and needs, as well as the problems they confront in online learning – to create policy. The majority of TVET students do not have access to a personal computer which is another barrier that prevents them from honing their fundamental technological abilities. In this regard, TVET institutions, in collaboration with other financial bodies, can provide student loans to buy a personal computer and other necessary equipment. Furthermore, the institution must be prepared in terms of technology (hardware and software) and staff in order to implement a blended learning strategy and must train students to use the technology when needed. The library may equip students and instructors with state-of-the-art formal and informal training resources. The goal is to provide the participants with the knowledge and abilities necessary to thrive in a blended learning setting.

This study also concludes that this BLRS scale could be used successfully to determine the level of readiness for blended learning among Bangladeshi TVET students irrespective of gender as well as previous academic performance. This scale identified that learners' propensity for blended learning is not affected by their gender, which demonstrates that TVET students of all genders in Bangladesh are equally enthusiastic about this innovative pedagogical approach. It is also interesting to identify that the student's previous academic accomplishment does not influence their readiness to engage in blended learning. This result signifies that students of all virtues have similar interests in this new concept and have experienced the same challenges and opportunities to encounter the next generation of teaching-learning methodologies.

## 8 Implications

The blended learning readiness scale reported in the current study appears to be more comprehensive than the blended learning readiness scale supplied by Tang [10]. It had some internal consistency reliability issues with few constructs. Besides, respondents to the questionnaire were never engaged in the blended learning session. Thus, this scale was not entirely applicable to Bangladeshi TVET students, specifically in the midst of the pandemic situation. Another purpose of this study is to determine whether Bangladeshi TVET students are prepared to participate in a blended learning setting. This study has the following implications: they will give course designers, students, TVET institutions, and the Bangladeshi government exposure so they can take drastic action to further increase student readiness for blended learning by considering six aspects of learning dominant factors and other contributing factors. This study has some practical implications for the head of TVET institutes, teachers, librarians, administrators, and policymakers. This study suggested that Bangladeshi TVET students lack basic skills in using technology. So, students need ICT training before engaging in the blended learning program. Some researchers, like Wang & Beasely [78] argue that using such measures would improve students' blended learning readiness and as well as academic performance.

Students reported feeling helpless to make changes to their learning environment and struggled with study management; therefore, the TVET teachers should make an extra effort to involve all students in task-based, online group discussions [76]. Thus, students would be better prepared for a mixed learning setting and more likely to participate actively in class. Teachers should plan engaging activities, such as having students vote on or comment on problems related to their online courses, to keep students engaged. Students with a low level of learner control can be helped by implementing a pre-test to determine where they currently stand academically and then being taught how to take charge of their own education by tailoring the curriculum and approach to their specific needs in a blended learning environment [2]. Collaboration between faculty and academic libraries can be a major success in improving blended learning-teaching dynamics. This article explored new approaches that academic librarians and instructors might use each other to help make the most of blended learning in the Bangladeshi TVET context.

## 9 Limitations and future research directions

This study has four research limitations. First, first-semester diploma engineering students of six TVET institutes were considered for this study. It can also be conducted on other semester students as well as on other TVET programs to generalize the findings. Secondly, the students were not given any technical assistance during the teaching-learning phase in the blended session. Providing technical help throughout the phases might result in a different understanding of blended learning on the part of participants. Thirdly, some items with less item loading were discarded, severely impacting a few dimensions and resulting in small effect sizes on those dimensions. However, a large sample size could minimize the effect size issue [64]. Lastly, expert reviews were taken from academicians with online teaching experience but limited blended learning teaching experience. Additionally, Students' responses to the Blended Learning Readiness Scale and comparable instruments were not collected simultaneously in this study. Correlations between the blended learning readiness scale and other analogous scales might be the subject of future studies to provide more concurrent proof of validity. The blended learning readiness scale's test-retest reliability is also an area that might be used for more investigation.

Topics for additional investigation are suggested by this study; for instance, once the current epidemic is gone, a mixed-methods study may be conducted. More than that, it would be worthwhile to do qualitative research to learn about the perspectives of students, teachers, and librarians. Studying the viewpoints of Bangladeshi TVET teachers and librarians who are already practicing blended teaching would be helpful in successfully implementing a blended learning program.

## Figures and Tables

**Table:** Underpinning Items of Readiness Dimensions after content validation

Dimension	Items No.	Items	Ref.
Attitude toward Learning Flexibility (LF)			
LF1	3	I would like to access the class materials on my own time	[70]
LF2		I would like to access the class materials at my own pace	[10]
LF3		I would like to access the class materials in my own space	[79]
Openness To (New) Technology (NT)			
NT1	5	I prefer technology while learning	
NT2		Access to all digital learning materials helps me understand my lesson precisely	[69] [79]
NT3		I would like to be in charge of my learning	
NT4		I would like to keep myself updated with new educational technology	[80]
NT5		I believe technology improves my quality of learning	
Attitude Toward Online Learning (OL)			
OL1	5	I believe online tasks help me build my learning capacities	[10] [79]
OL2		I am comfortable with self-directed online learning	[70]
OL3		I do not feel isolated in an online classroom	
OL4		I believe online learning helps me to prepare well for my future endeavours.	[10]
OL5		I prefer an online platform to communicate with other teachers and students	
Attitude Toward Face-To-Face Classroom (CL)			
CL1	5	I prefer to learn in the face-to-face classroom environment	[66]
CL2		I learn better in a teacher-directed face to face classroom	[66]
CL3		I believe face-to-face learning develops my interpersonal and team-building skills	[80]
CL4		I prefer immediate feedback from the teacher	[79]
CL5		I prefer learning through collaboration with other people in Face-To-Face Classroom	[79]
Basic skills in Using Technology (BST)			
BST1	4	I have basic skills in using technology	[70]
BST2		I can understand the online instruction for assignments/quizzes/tutorials	
BST3		I can manage unwanted situations and download the learning materials	
BST4		I prefer browsing the website and various new applications	[66]
Attitude Toward Study Management (SM)			
SM1	3\	I believe a change in the teaching-learning environment has a positive influence on my learning ability	
SM2		Online discussion and collaboration help me to learn efficiently	[10]
SM3		I believe online learning helps me organize my time better	[69]
Readiness Toward Blended Learning (BL)			
BL1	4	I am more comfortable with blended learning than face-to-face learning	[10]
BL2		I want to attend courses that offer blended learning	[69]
BL3		I believe a blended learning environment improved my learning capacity	
BL4		I am open to new ideas and concepts	
